# Selected Physicochemical Properties of Diamond Like Carbon (DLC) Coating on Ti-13Nb-13Zr Alloy Used for Blood Contacting Implants

**DOI:** 10.3390/ma13225077

**Published:** 2020-11-11

**Authors:** Magdalena Antonowicz, Roksana Kurpanik, Witold Walke, Marcin Basiaga, Jozef Sondor, Zbigniew Paszenda

**Affiliations:** 1 Department of Biomaterials and Medical Devices Engineering, Faculty of Biomedical Engineering, Silesian University of Technology, 41-800 Zabrze, Poland; witold.walke@polsl.pl (W.W.); marcin.basiaga@polsl.pl (M.B.); zbigniew.paszenda@polsl.pl (Z.P.); 2 Department of Biomaterials and Composites, Faculty of Materials Science and Ceramics, AGH University of Science and Technology, 30-059 Kraków, Poland; roksana.kurpanik@gmail.com; 3 LISS a.s, Dopravni 2603, 756 61 Rožnov pod Radhoštěm, Czech Republic; j.sondor@liss.cz

**Keywords:** Ti-13Nb-13Zr alloy, DLC layer, surface wettability, EIS, potentiodynamic test, scratch test

## Abstract

Despite high interest in the issues of hemocompatibility of titanium implants, particularly those made of the Ti-13Nb-13Zr alloy, the applied methods of surface modification still do not always guarantee the physicochemical properties required for their safe operation. The factors that reduce the efficiency of the application of titanium alloys in the treatment of conditions of the cardiovascular system include blood coagulation and fibrous proliferation within the vessel’s internal walls. They result from their surfaces’ physicochemical properties not being fully adapted to the specifics of the circulatory system. Until now, the generation and development mechanics of these adverse processes are not fully known. Thus, the fundamental problem in this work is to determine the correlation between the physicochemical properties of the diamond like carbon (DLC) coating (shaped by the technological conditions of the process) applied onto the Ti-13Nb-13Zr alloy designed for contact with blood and its hemocompatibility. In the paper, microscopic metallographic, surface roughness, wettability, free surface energy, hardness, coating adhesion to the substrate, impendence, and potentiodynamic studies in artificial plasma were carried out. The surface layer with the DLC coating ensures the required surface roughness and hydrophobic character and sufficient pitting corrosion resistance in artificial plasma. On the other hand, the proposed CrN interlayer results in better adhesion of the coating to the Ti-13Nb-13Zr alloy. This type of coating is an alternative to the modification of titanium alloy surfaces using various elements to improve the blood environment’s hemocompatibility.

## 1. Introduction

Implant hemocompatibility is a complex issue that determines its usability in the human body [1,2,3]. These are broad terms that include many factors, including mechanical, physical, chemical, and electrochemical properties, both of the biomaterial itself and the finished implant [4,5,6]. Another essential factor that impacts hemocompatibility is surface preparation [7,8]. The morphology and topography of the surface layer, which is the first to make contact with the tissue environment, is vital in the context of implant acceptance or rejection [9,10]. Improper selection of its stereological and physicochemical properties may result in a chronic inflammatory reaction, bacterial biofilm formation, or cytotoxic activity. The ions released from the surfaces of medical products may cause allergic, mutagenic and oncogenic reactions in the human body. In particular, their accumulation in filtering organs, such as the pancreas, kidneys, spleen, have an impact on the impairment of this type of organs [11,12,13]. The implants used in the treatment of cardiovascular system conditions are exposed to the processes of blood coagulation and fibrous proliferation within the vessel’s internal walls. This results from their surfaces’ physicochemical properties not being adapted to the specifics of the circulatory system [14,15].

Despite high interest in the issues of the hemocompatibility of titanium implants, the applied methods of surface modification still do not always guarantee the biological and physicochemical properties required for their safe operation. Due to good mechanical properties and inertness, diamond like carbon (DLC) coatings are a promising selection for applications in the circulatory system, as a coating on heart valves or artificial heart components [16]. As reported in the literature [17], their application significantly reduces the friction coefficient and increases surface hardness. Furthermore, the main component of coatingscarbonis one of the building elements of the human body, which reduces the risk of toxic reactions. Due to the character of the operating environment, such as in the case of a heart assist centrifugal pump, particular emphasis is on mechanical properties (hardness, friction coefficient) and physical properties (wettability) of the surface layer [18,19]. Moreover, medical products in contact with blood should be characterised by proper surface roughness, which directly impacts on the coagulation process. For the coating to serve its purpose properly, its interaction with the morphology and proper adhesion to the substrate on which it is applied, is essential. At present, the purpose of studies is to propose such coating with carbon content, as well as surface treatment of the substrate, which under technological conditions will ensure its proper adhesion, thus providing efficient protection against a corrosive environment—blood [20,21,22]. Therefore, to improve the adhesion of carbon coatings, interlayers are increasingly used that create a strong bond between the coating and the substrate material. This layer has different physicochemical properties and proper corrosion resistance. For example, if silicon is used as the interface, environmental conditions must be considered. The research in [23], proved that the containing adhesion layer shows inadequate resistance to lymph reactions. In addition, the proteins and cells in it—in the case of a breach of the DLC coating—create fissure conditions. Then, the corrosion they cause contributes to coating delamination from the surface. Furthermore, through friction and blood environment impact, the inert coating could react with water and oxygen particles. Therefore, another important factor is the composition of the layer. Hydrogen content could influence the friction coefficient by increasing it in a humid environment or decreasing it [24]. As shown by another study [25], the durability of the DLC coating in the lymph environment is ten times lower than in a dry environment. Thus, admixtures of elements reacting with oxygen and water are used, i.e., Si, F, B, and N. They have an influence on the friction coefficient stability [26,27]. The quality of the obtained DLC coatings also depends on the method of application [28]. One of the most common is plasma-enhanced chemical vapor deposition (PECVD). This type of technology can be used for implants with simple, uncomplicated shapes of implants, such as heart valve rings. The most important advantage of this method is that even coating application is obtained at a lower process temperature [29]. Thus, this work includes an attempt at determining the impact of the DLC coating (shaped by the technological conditions of the PECVD process) applied onto Ti-13Nb-13Zr alloy designed for contact with blood on the improvement of the physicochemical parameters that define the hemocompatibility of the alloy.

## 2. Materials and Methods

The specimens included Ti-13Nb-13Zr alloy samples in the form of discs with a diameter of d = 14 mm and the thickness of g = 3 mm. The chemical composition of the Ti-13Nb-13Zr alloy is shown in Table 1. In order to reveal the structure, samples were subjected to the process of etching in 8% hydrofluoric acid (HF) for the time of t = 6–8 s. Observations were made using the optical microscope ZEISS Observer Z1 (Zeiss, Oberkochen, Germany), in a reversed system, in the magnification range of 100–1000×.

**Figure 1:**
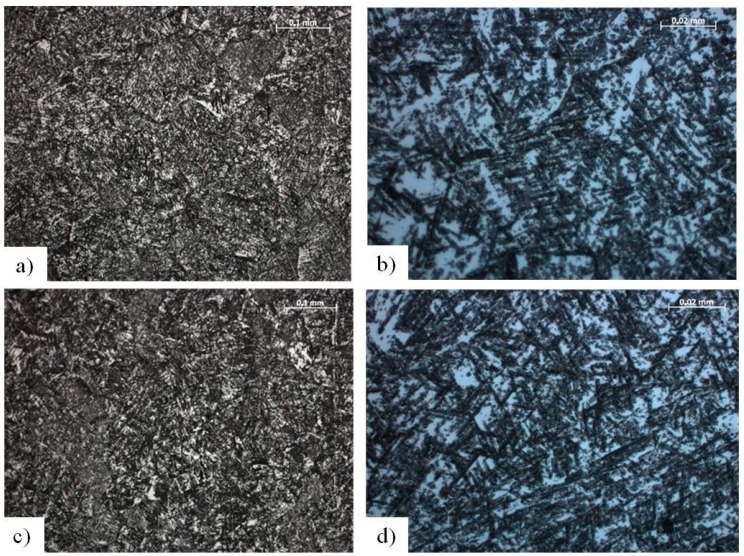
The microstructure of the Ti-13Nb-13Zr alloy (**a**) Polished longitudinal section, mag. 200×; (**b**) Polished longitudinal section, mag. 1000×; (**c**) Polished transverse section, mag. 200×; (**d**) Polished transverse section, mag. 1000×.

Figure 1 shows the microscopic images of the Ti-13Nb-13Zr alloy. A two-phase microstructure was found, β + α’ (predominant phase β with a share of fine needle martensite of phase α’), with varied grain size and visible deformation twins and annealing twins. This type of structure is characteristic of the Ti–13Nb–13Zr alloy after cold plastic deformation. Grain size was determined using the comparative method based on ASTM E112: Standard Test Methods for Determining Average Grain Size [31], is within the range of reference number G = 2–6.

Concerning the chemical composition and structure, the alloy meets the recommendations of ASTM F1713–08(2013)–Standard Specification for Wrought Titanium-13Niobum-13Zirconium Alloy for Surgical Implant Applications [30].

The samples delivered were divided into two test groups that were subjected to different methods of surface treatment, i.e.,:

1. Mechanical treatment by abrasive blasting (sand-blasting with SiO_2_ beads with a grit of 50 µm, t = 5 min, p = 4 atm),
2. Mechanical treatment (polishing using a SiO_2_ solution on abrasive paper with a grit of 1200 grains/mm^2^)

**Figure 2:**
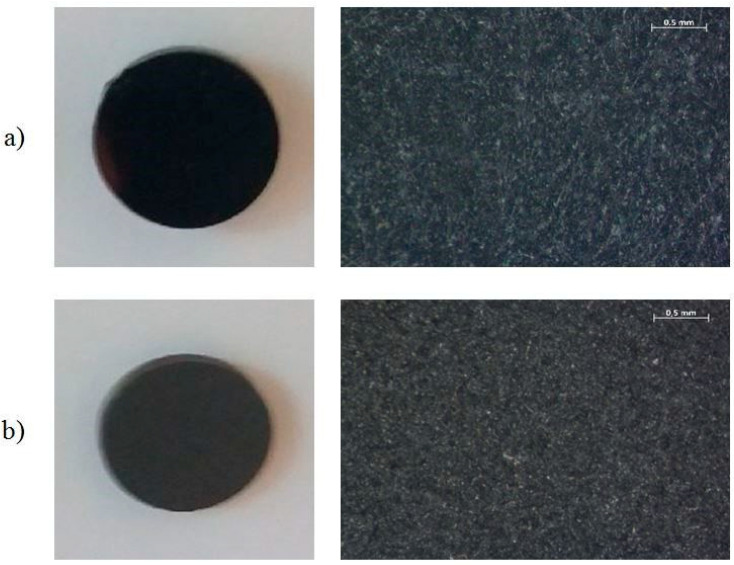
Sample surface: (**a**) polished, (**b**) subject to treatment by abrasive blasting with sand with a grit of 50 µm, with diamond like carbon (DLC) coating.

On the prepared surfaces, coating type a: C–H(Si) with a thickness of g = 1000 nm with an interlayer of CrN (g = 20 nm) was applied using the PECVD method. The process temperature was 240 °C in CH_4_ active medium, voltage E= −80 V, pressure p= 0.02 Pa. Figure 2 shows the surfaces of samples after the DLC coating application. The coating was applied in collaboration with LISS Group, Rožnov pod Radhoštěm, Czech Republic.

At the final stage, all samples were subjected to the process of medical sterilization in an autoclave—with pressurized water vapour (T = 135 °C, p = 2.1 bar, t = 7 min).

### 2.1. Surface Morphology

Surface roughness measurements were made using the contact method as per PN–ISO 4288:1997 [32]. Used for this purpose was the diamond-tipped mechanical contact profilometer Sutronic 3+ by Taylor-Hobson (Sutronic 3+, Taylor-Hobson, Leicester, United Kingdom), Five measurements per sample were made in two perpendicular directions, then the arithmetic mean was calculated. The test was performed by placing the diamond tip of the profilometer head on the sample surface. The measurement was completed on a measurement section lc = 0.8 mm with a precision of ±0.01 µm. During the measurement, the value of surface roughness Ra was determined, which specifies the mean arithmetic deviation of the mean line profile.

### 2.2. Surface Wettability

To determine the selected sample surface wettability, tests were made for contact angle tests using the sitting drop method and free surface energy (SEP) using the Owens–Wendt method. The measurements of the contact angle of each surface were made using distilled water (θw) (Avantor mPerformance Materials Poland, Gliwice, Poland). and diiodomethane (θd) (Merck Group, Darmstadt, Germany) at room temperature T = 23 °C on a test bench consisting of goniometer SURFTENS UNIVERSAL by OEG (OEG Gesellschaft fur Optik, Elektronik & Geratetechnik mbH, Frankfurt (Oder), Germany) and a computer with Surftens 4.5 software for analysing the recorded drop image. Five drops of distilled water and diiodomethane with a volume of 1.5 mm^3^ were applied onto each sample’s surface. The measurement was started 10 s from the application. The duration of one measurement was 60 s, with a sampling frequency of 1 Hz. Based on the tests, mean values of the contact angle θavg and free surface energy γs were determined. The values of free surface energy (SEP) assumed for calculations and their components (polar and dispersive) were, respectively: for distilled water—γsp = 51.0 [mJ/m^2^] and γsd = 21.8 [mJ/m^2^]; for diiodomethane—γsp = 6.7 [mJ/m^2^] and γsd = 44.1 [mJ/m^2^].

### 2.3. Mechanical Properties

#### 2.3.1. Hardness

**Figure 3:**
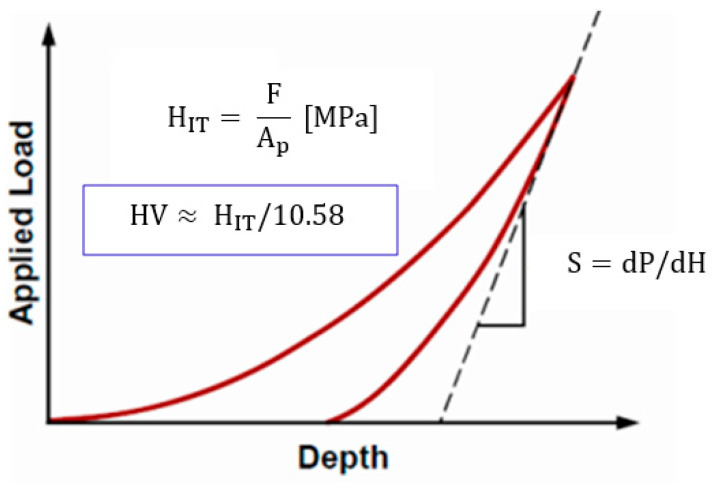
Instrumental microhardness test—the Oliver and Pharr method.

Tests of hardness (microhardness, nanohardness) were made both for the substrate material and the coating. Microhardness tests of the Ti–13Nb–13Zr alloy were conducted using the Vickers method, with a force loading of F = 0.9807 N. 10 measurements were made for both the longitudinal and polished transverse section of the Ti-13Nb-13Zr alloy. For testing, hardness tester Dura Scan by Struers (Dura Scan, Struers, Cleveland, OH, USA) was used. On the other hand, nanohardness, measurements of the coating formed on the Ti–13Zn–13Nb alloy surface using the Olivera and Phara method (Figure 3), were made on an open platform equipped with Micro-Combi-Tester by CSM Instruments (Micro-Combi-Tester, CSM instruments a company of Anton Paar, Peseux, Switzerland), using a Vickers penetrator. The purpose of this test was to determine the instrumental hardness as a function of penetration depth. The load increase and decrease rate was 3000 nm/min, while the sample holding time under maximum pressure was 5 s. The penetrator load value was the output. Microhardness was measured at the following penetrator depth values: 250 nm, 500 nm, 1000 nm, 1500 nm, 2000 nm.

#### 2.3.2. Scratch Test

The adhesion tests of the DLC coatings applied onto the Ti–13Nb–13Zr alloy were performed using the scratch test method as per PN-EN ISO 20502:2016-05 [33]. The measurements were made using an open platform equipped with a Micro-Combi-Tester by CSM (Micro-Combi-Tester, CSM instruments a company of Anton Paar, Peseux, Switzerland). The test consisted of making a scratch using a Rockwell diamond cone, acting as the penetrator, with a gradual increase in the normal force loading the penetrator. The critical force, which is the adhesion measure, is the lowest normal force that causes loss of adhesion of the coating to the substrate. The value of the critical force Lc was evaluated based on the friction force (FT) and friction coefficient (μ) and observations made with the optical microscope, which is an integral part of the platform. Measurements were made with the loading force increasing from 0.03 to 30 N. Measurements were made with the following parameters: load increase rate 10 N/min, bench travel speed 1 mm/min, scratch length ~3 mm.

### 2.4. Electrochemical Properties

#### 2.4.1. Potentiodynamic Test

The pitting corrosion resistance was tested using the potentiodynamic method, based on recording polarisation curves using a bench consisting of a test system VoltaLab PGP201 by Radiometer (Radiometer, Villeurbanne Cedex, Lyon, France): reference electrode (saturated calomel electrode SCE type KP-113), auxiliary electrode (platinum electrode type PtP-201), anode (test sample) and a PC with VoltaMaster 4 software 

Pitting corrosion resistance was tested following the recommendations of PN-EN ISO 10993-15 [34] in a solution simulating artificial plasma with the following chemical composition and concentration: CaCl_2_–0.2 g/L, NaCl–6.8 g/L, KCl–0.4 g, MgSO_4_–0.1 g/L, NaHCO_3_–2.2 g/L, Na_2_HPO_4_–0.126 g/L and NaH_2_PO_4_–0.026 g/L at the temperature of T = 37 ± 1 °C, pH = 7.0 ± 0.2. First, the opening potential E_OCP_ was determined under de-energised conditions. Then, polarisation curves were recorded, starting from the initial potential value E_START_ = E_OCP_ − 100 mV, with a potential change rate of v = 0.16 mV/s in the anode direction. Samples were polarised up to the current density of i = 1 mA/cm^2^. After reaching this value, the polarisation direction was changed to record the return curve. Thus recorded curves were the basis for determining characteristic values to describe corrosion resistance, i.e., corrosion potential—E_corr_, polarisation resistance R_p_ and corrosion current i_kor_ (Stern method).

#### 2.4.2. Electrochemical Impedance Spectroscopy Test

Electrochemical Impedance Spectroscopy (EIS) tests were made to obtain additional information on the surface’s physicochemical properties and the character of the coating formed on it. Measurements were made using test system AutoLab PGSTAT 302N (Metrohm autolab, Utrecht, The Netherlands), equipped with module FRA2 (frequency response analysis). The electrode system used was identical with that used in the potentiodynamic measurements. Through testing, impedance spectra of the circuit were determined and the obtained measurement data were adjusted to an equivalent electrical circuit. EIS tests were made like potentiodynamic tests, in an artificial plasma solution. During the measurement, circuit impedance variation was recorded as a function of alternating current pulse frequency within the frequency range of 10^4^–10^−3^ Hz. The amplitude of the activating sinusoidal voltage was 10 mV. Through measurements, impedance spectra of the circuit were determined and presented as Nyquist and Bode diagrams. Next, using the least squares method, equivalent electrical circuits were adjusted to them. Thus determined circuits were used to determine the values of resistance R and capacity C of the circuits analysed. At the last stage, test results for samples with the DLC coating were compared to the values obtained for samples in initial condition.

## 3. Results

### 3.1. Surface Roughness Test

**Figure 4:**
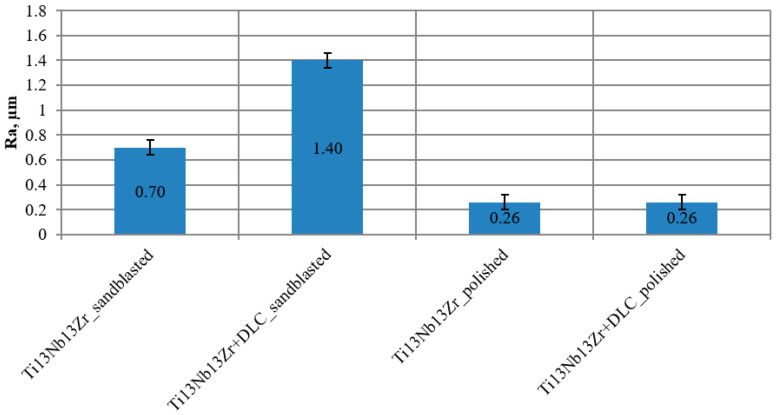
Mean surface roughness values.

Figure 4 shows mean values of roughness Ra with standard deviation. For samples in the initial condition, this value was 0.26 µm for the polished surface and 0.70 µm for the sand-blasted surface. In case of samples with the DLC coating applied, this value did not change for the polished surface, but increased two times for a sand-blasted surface. The last variant also featured the broadest standard deviation.

### 3.2. Tests of Contact Angle and Free Surface Energy

**Figure 5:**
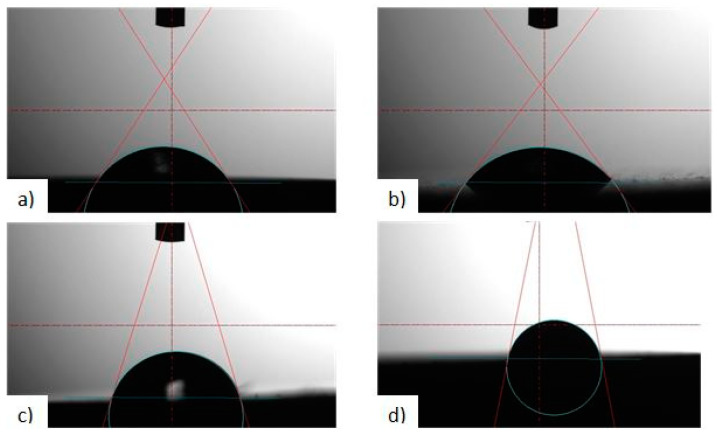
Example contact angle measurement for a sample: (**a**) polished, initial state, (**b**) sand-blasted, initial state, (**c**) polished, with the DLC coating, (**d**) sand-blasted with the DLC coating.

Based on the obtained wettability and surface energy results, an increase in the contact angle was found for all variants of surface modifications in relation to the initial state. The mean value for the polished and sand-blasted sample in the initial state is 60.79° and 55.03° (distilled water), and 58.13° and 51.15° (diiodomethane), respectively. Moreover, the mean value of the analysed parameter does not differ significantly between the two variants of surface modification. Thus, the application of the DLC coating resulted in an increase in the contact angle, visible above all for the sample subject to abrasive blasting treatment. This value was up to 73.75° (distilled water), 56.58° (diiodomethane) for polished surface and 91.53° (distilled water), 67.31° (diiodomethane) for the sand-blasted surface. Example photos of distilled water drops applied onto the Ti-13Nb-13Zr titanium alloy in initial state and after the application of the DLC layer during testing are shown in Figure 5. The contact angle and surface energy results for all evaluated surfaces are shown in Table 2.

### 3.3. Hardness Test

#### 3.3.1. Microhardness Test

**Figure 6:**
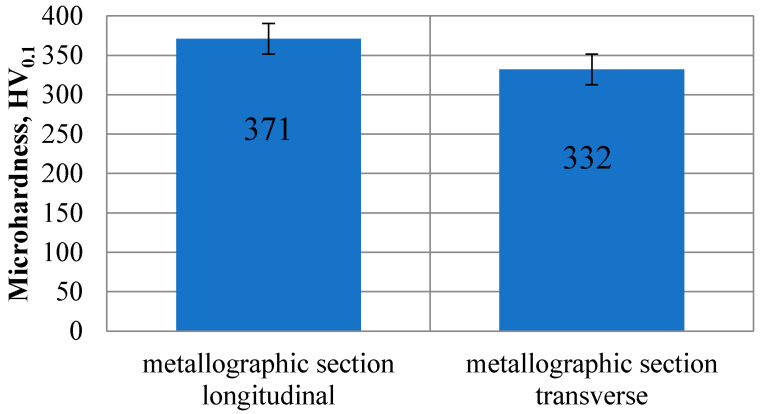
Microhardness measurement results for the Ti-13Nb-13Zr alloy.

Figure 6 shows the mean values obtained through microhardness testing using the Vickers method. In case of a longitudinal polished section this value was constant at approximately 371 HV_0,1_. On the other hand, in case of a transverse polished section, these values were variable depending on the test point, from 260 to do 444 HV_0,1_. The slight difference in microhardness values obtained between the longitudinal and transverse sample taken from the rod is caused by the manufacturing—shaping technology (drawing process).

#### 3.3.2. Nanohardness Test

**Figure 7:**
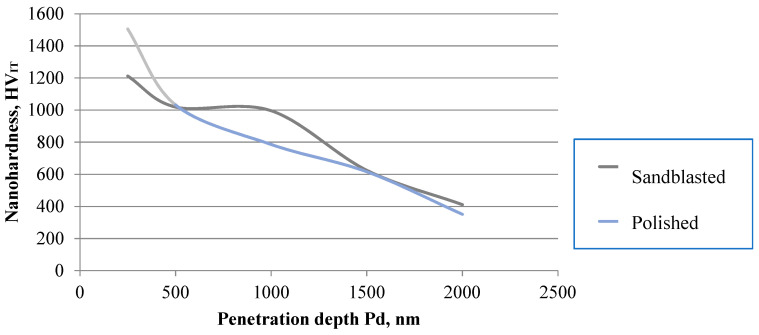
Depth profile of microhardness for the DLC coating applied onto the Ti-13Nb-13Zr alloy.

Nanohardness test results for the DLC coating applied onto the Ti–13Nb–13Zr alloy are shown in Figure 7. Based on the obtained data, it can be stated that the DLC coating features a significantly higher hardness in relation to the substrate. This value is 1500 HV for a coating applied on a polished surface and 1200 HV for a substrate subject to abrasive blasting treatment.

#### 3.3.3. Test of Adhesion of the Coating to the Substrate

**Figure 8:**
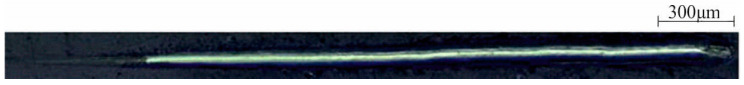
Test results of adhesion of the DLC coating to the Ti-13Nb-13Zr alloy—polished.

**Figure 9:**
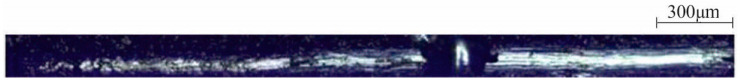
Test results of adhesion of the DLC coating to the Ti-13Nb-13Zr alloy—sand-blasted.

Table 3 includes the values of critical force L_C1_ (first cracking), L_C2_ (first delamination), and L_C3_ (full delamination). Figure 8 and Figure 9 show the curves recorded during the test with a graphical image of the mark. For the polished sample, L_C3_ is 5.53 N and it is almost two times higher than for the sample subject to abrasive blasting treatment (2.81 N). However, the difference between force L_C2_ and L_C3_ is ~1 N for the first variant and ~2 for the second variant, respectively.

#### 3.3.4. Potentiodynamic Tests

**Figure 10:**
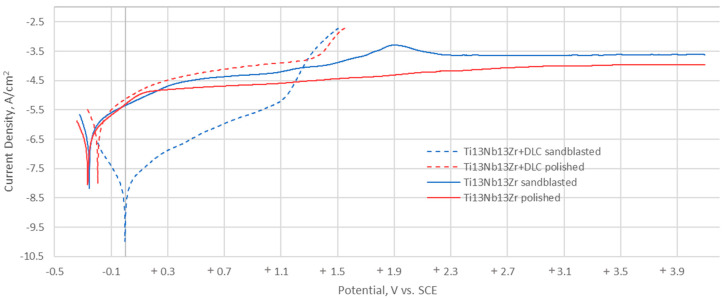
Typical polarisation curves for Ti-13Nb-13Zr and Ti-13Nb-13Zr+DLC.

Figure 10 shows the polarisation curves representing the change in density of the corrosion current as a function of potential changes, based on which basic parameters were determined to describe the resistance of test samples to pitting corrosion (Table 4).

The two variants show different corrosion current densities (0.479 µ/cm^2^ for the sand-blasted surface and 0.297 µA/cm^2^ for the polished surface), however this difference is insignificant in case of corrosion potential (approximately 10 mV). It can be noted that their shape differs from the shapes obtained for samples without coating. The two variants show a significant difference in corrosion potential, which is 1 mV for the sand-blasted surface and 195 mV for the polished surface. However, the differences in the pitting and repassivation potential are insignificant, 38 mV and 75 mV, respectively.

#### 3.3.5. Tests with Electrochemical Impedance Spectroscopy

**Figure 11:**
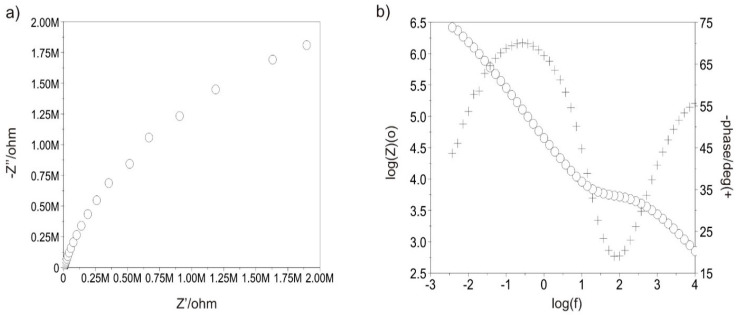
Polished sample with the DLC coating: (**a**) Nyquist diagram (**b**) Bode diagram. ◦: log(Z); +: -phase/deg.

**Figure 12:**
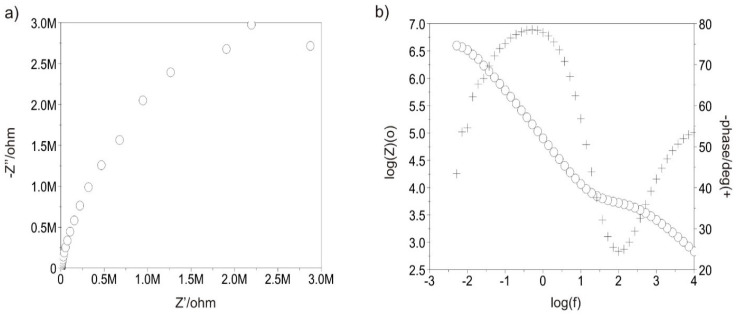
Sand-blasted sample with the DLC coating: (**a**) Nyquist diagram and (**b**) Bode diagram. ◦: log(Z); +: -phase/deg.

Figure 11 and Figure 12 show the example impedance spectra recorded for all variants of test samples. Based on the determined Nyquist diagrams, it can be stated that the surface of each test sample shows a layer character similar to capacitive. This is proved by the plot shape, which is close to a semi-circle, and log inclination |Z| in the entire range of frequency changes, which is approximately −1. On the other hand, the Bode diagram log|Z|—log(f) indicates the presence of a double layer for the polished sample in initial state. The impedance spectra, which were only obtained for the sample after the polishing process were interpreted by comparison with an equivalent electrical circuit. The comparison indicates the presence of two sublayers: a compact internal layer and porous external layer (two time constants visible in the plot), where Rs is the resistance of the electrolyte, R_pore_ is the resistance of the electrolyte in pores, CPE_pore_ is the capacity of the double layer (porous, surface layer), and R_ct_ and CPE_dl_ are the resistance and capacity of the passive layer. This equivalent electrical circuit can be described using the following relationship (1), which defines its outcome impedance:

(1) $$Z=\;R_{s}+\;\frac{1}{\frac{1}{R_{pore}}+\;Y{\left(j\omega \right)}^{n}}+\frac{1}{\frac{1}{R_{ct}}+Y\;{\left(j\omega \right)}^{n}}$$

**Figure 13:**
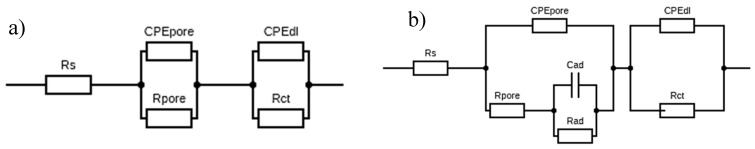
Equivalent electrical circuits of corrosion systems for: (**a**) sample in initial state, polished, (**b**) samples with the DLC coating and sample in initial state, sand-blasted.

The use of two solid phase elements in the equivalent electrical circuit had a beneficial influence on the quality of adjustment of the experimentally obtained curves. However, in the case of both variants with the DLC coating applied and the sample in initial condition subject to abrasive blasting treatment, the presence of an additional absorpton layer was found, characterised by resistance R_ad_ and capacity C_ad_—Figure 13.

The electrical parameters obtained from equivalent electrical circuits created based on the obtained impedance spectra are included in Table 5. Based on impedance values |Z| (of the order of 106 Ω) and the obtained electrical parameters, it can be noted that the DLC coating deposition influenced the increase in the charge transfer resistance, which in turn proves high dielectric properties of the surface, which is advantageous.

## 4. Discussion

The research conducted by the authors showed that the method of substrate preparation influences the physicochemical properties of the surface layer with the DLC coating. It was found that the mechanical polishing process that enabled the obtainment of surface roughness recommended for products for contact with blood also influenced the adhesion of the coating to the titanium substrate. The TiO_2_ surface layer obtained through polishing, as a result of the self-passivation process, constituted an additional sublayer for the CrN interlayer, which caused the adhesion and diffusion character of DLC coatings. The depth profile made for nanohardness measurements of the surface layer had a linear character. Hardness values from the substrate (core) to the DLC coating were found to decrease evenly. The diffusion character was also confirmed in scratch tests. This is confirmed by the higher critical force L_C3_ obtained for the polished sample, compared to the sand-blasted sample.

The method of substrate preparation proposed by the authors, which includes mechanical polishing, will also enable the obtainment of suitable surface roughness, acceptable for implants having contact with blood. On the other hand, the results obtained during surface roughness measurements after the application process of DLC coatings prove that these only inherit the substrate in case of surface treatment (mechanical polishing). However, in the case of samples subject to abrasive blasting treatment, the value of Ra is significantly higher after the application of the DLC coating. The high standard deviation indicates greater surface irregularity in relation to the initial state. In addition, the obtained results show the impact of surface development on the character of the coating—more adhesive in this case. This is proved by the bilinear course recorded during nanohardness tests (depth profile) and lower critical force L_c2_. The physical properties of the surface layer also contribute to improved hemocompatibility. The contact of the biomaterial with artificial plasma nduces processes that lead to clot formation. Surfaces characterised by high surface energy cause a higher absorption of proteins and the resulting cell adhesion. An inverse phenomenon occurs for materials with low surface energy [35,36,37,38,39]. Thus, at the following state of work, the authors researched the contact angle and free surface energy. Contact angle measurements showed that in the case of samples without coating, values were within the characteristic range for surfaces with a moderate hydrophilic character. The coating applied, both onto samples with surfaces subject to mechanical polishing and abrasive blasting treatment, increased the contact angle and thus changed the character from hydrophilic to hydrophobic. This is advantageous for implants used in the circulatory system, for which low protein absorption is desirable to reduce the blood clotting process. The analysed coating, regardless of the substrate preparation method, was characterised by low free surface energy compared to samples without coating, which indicated weak adhesion properties of blood morphology components, as well as a relatively high resistance to the penetration of corrosive compounds with water into the DLC coating structure. Coating tightness, and thus its role in reducing direct contact of the titanium substrate with blood, is an important factor influencing corrosion resistance. In turn, corrosion resistance has a direct impact on hemocompatibility. Thus, as the final stage of the study, the authors evaluated DLC coatings’ electrochemical properties using potentiodynamic and impedance methods. The conducted potentiodynamic tests proved that regardless of the method of surface modification, the Ti–13Nb–13Zr alloy is fully resistant to pitting corrosion within the potential range of up to +4 V. On the other hand, the measurements conducted for DLC coatings demonstrated a contribution to the increase in the corrosive potential value, which is advantageous. Although in the case of DLC coatings, hysteresis loops were recorded, indicating corrosion process initiation. It was located at the potential values that do not naturally occur in the human body, above +1200 mV. The perfect passivation area is in the range of −195 mV to +1103 mV for polished substrate and −1 mV to +1028 mV for sand-blasted substrate.

Supplementary tests conducted using electrochemical impedance spectroscopy confirmed the DLC coating’s efficiency for protecting the polished substrate from corrosive environmental impact. The recorded charge transfer resistance R_ct_ was 4.88 MΩ cm^2^, higher than that obtained for the Ti-13Nb-13Zr titanium alloy. To conclude, the research conducted clearly proved that the DLC coating applied using the PECVD method (under defined technological conditions) with the CrN interlayer for the Ti-13Nb-13Zr alloy subject to the mechanical polishing process, ensures an advantageous set of physicochemical properties, which improves parameter values. Thus, it is an alternative to the modification of Ti-13Nb-13Zr alloy surfaces using various elements and application methods designed to improve the hemocompatibility in the blood environment.

## 5. Conclusions

Based on the research, the following generalisations were formulated:

- The passive layer formed spontaneously on the Ti-13Nb-13Zr alloy during initial surface treatment (mechanical polishing and sandblasting) improves corrosion resistance. Additionally, the resulting low contact angle indicates its hydrophilic character, which is not desirable.
- DLC coating roughness tests showed a tendency to inherit the stereometric parameters of the surface of the tested titanium substrate only in the case of mechanical polishing (treatment preceding the application process (Ra < 0.30 μm)).
- The application of a compact DLC coating with the CrN interlayer by the PECVD method in the conditions proposed by the authors of the study effectively reduces the migration of Ti, Nb, Zr ions, which was confirmed in potentiodynamic and impedance tests. It also improves the Ti-13Nb-13Zr alloy surface’s wettability, changing its character from hydrophilic to hydrophobic.
- The deep nanohardness profile that was determined, clearly showed that the application of the CrN interlayer for the substrate after mechanical polishing causes a linear course of changes in the value, thus ensuring very good adhesion of the DLC coating, which was also confirmed in the studies scratch-test.
- Through the adhesion study, the influence of the Ti-13Nb-13Zr alloy surface preparation was shown. It was found that the DLC layer deposited on the polished surface of the Ti-13Nb-13Zr alloy showed the best adhesion, as evidenced by the values of the Lc parameter.

The method of preparing the substrate definitely has an impact on the physicochemical properties of the surface layer with the DLC coating.

## Figures and Tables

**Table 1:** Chemical composition of the Ti–13Nb–13Zr alloy.

Type of Analysis	Chemical Composition (wt. %)							
	Nb	Zr	Fe	C	N	O	H	Ti
ASTM F-1713 [30]	12.5–14.0	12.5–14.0	<0.25	<0.08	<0.05	<0.15	<0.015	rest
Certificate	13.5	13.5	0.05	0.04	0.013	0.11	0.04	rest

**Table 2:** Results of the wettability and surface energy.

Sample	Contact Angle θ_avr_. °		Polar Component γ_s_^p^.mJ/m^2^	Dispersion Component γ_s_^d^.mJ/m^2^	Surface Energy (SEP) γ^s^. mJ/m^2^
	Distilled Water	Diiodomethane			
Polished_Ti-13Nb-13Zr initial state	60.79 ± 4.64	58.13 ± 0.27	25.51 ± 0.13	15.02 ± 0.16	40.52 ± 0.02
Polished_Ti-13Nb-13Zr_DLC	73.75 ± 1.74	56.58 ± 0.75	12.60 ± 0.26	20.68 ± 0.51	33.28 ± 0.25
Sandblasted _Ti-13Nb-13Zr_initial state	55.03 ± 1.22	51.15 ± 0.37	28.08 ± 0.18	17.29 ± 0.21	45.38 ± 0.03
Sandblasted _Ti-13Nb-13Zr_DLC	91.53 ± 3.94	67.31 ± 2.27	4.04 ± 0.50	20.42 ± 1.68	24.47 ± 1.18

**Table 3:** Values of critical force L_C_ for the evaluated variants.

Sample	Failure of the Layer	Average Critical Value of Registered Indenter Load, F, N
Polished_Ti-13Nb-13Zr_DLC	First cracking Lc_1_	−
	First delamination Lc_2_	4.56
	Full delamination Lc_3_	5.53
Sandblasted _Ti-13Nb-13Zr_DLC	First cracking Lc_1_	−
	First delamination Lc_2_	1.28
	Full delamination Lc_3_	2.81

**Table 4:** Results of potentiodynamic tests.

Sample		E_ocp_ (mV)	E_b_ (mV)	E_cp_ (mV)	E_corr_ (mV)	R_p_ (MΩ *cm^2^)	i_corr_ (µA/cm^2^)
Ti-13Nb-13Zr	Sandblasted	−224	−	−	−255	0.054	0.479
	Polished	−243	−	−	−266	0.088	0.297
Ti-13Nb-13Zr+DLC	Sandblasted	+97	+1358	+1028	−1	0.038	0.765
	Polished	−169	+1397	+1103	−195	0.031	0.832

**Table 5:** EIS results.

Sample		Ti-13Nb-13Zr Polished	Ti-13Nb-13Zr Sandblasted	Ti-13Nb-13Zr + DLC Polished	Ti-13Nb-13Zr + DLC Sandblasted
R_s_ (Ω)		17	17	17	17
CPE_pore_	y_0_ (Ω^−1^∙cm^−2^∙s^−n^)	0.2954×10^−4^	0.1325×10^−3^	0.3277×10^−6^	0.4046×10^−6^
	n	0.85	0.86	0.77	0.75
R_pore_ (kΩ cm^2^)		2.35	1.33	4.97	4.89
CPE_dl_	y_0_ (Ω^−^^1^∙cm^−2^∙s^−n^)	0.1952×10^−3^	0.1337×10^−3^	0.5596×10^−5^	0.2614×10^−5^
	n	0.83	0.76	0.82	0.88
R_ct_ (MΩ cm^2^)		0.046	0.058	4.880	7.080
C_ad_ (µF)		30	662	48	-
R_ad_ (kΩ cm^2^)		28.00	2.99	60.20	-
E_OCP_ (mV)		−330	−273	58	41
